# Design for Robustness: Bio-Inspired Perspectives in Structural Engineering

**DOI:** 10.3390/biomimetics8010095

**Published:** 2023-02-26

**Authors:** Foad Kiakojouri, Valerio De Biagi, Lorenza Abbracciavento

**Affiliations:** Department of Structural, Geotechnical and Building Engineering (DISEG), Politecnico di Torino, 10129 Torino, Italy

**Keywords:** structural robustness, local damage, complexity, compartmentalization, bio-inspiration

## Abstract

Bio-inspired solutions are widely adopted in different engineering disciplines. However, in structural engineering, these solutions are mainly limited to bio-inspired forms, shapes, and materials. Nature is almost completely neglected as a source of structural design philosophy. This study lists and discusses several bio-inspired solutions classified into two main classes, i.e., compartmentalization and complexity, for structural robustness design. Different examples are provided and mechanisms are categorized and discussed in detail. Some provided ideas are already used in the current structural engineering research and practice, usually without focus on their bio-analogy. These solutions are revisited and scrutinized from a bio-inspired point of view, and new aspects and possible improvements are suggested. Moreover, novel bio-inspired concepts including delayed compartmentalization, active compartmentalization, compartmentalization in intact parts, and structural complexity are also propounded for structural design under extreme loading conditions.

## 1. Introduction

Biomimetics is a growing design approach in various fields of engineering. It consists of emulating, i.e., being inspired by principles, structures, or other solutions found in nature. Basically, it can be argued that the never-ending evolution that takes place in nature leads to alternative but equally effective solutions to many problems. To export solutions from nature to engineering, problem-based or solution-based approaches are possible. The former refers to a top-down approach to search for a bio-inspired solution for a particular engineering problem. The latter, on the contrary, is bottom-up, that is taking inspiration from nature for a new engineering design [1].

Bio-inspired solutions have become very popular nowadays and have been widely used in the structural engineering realm. Three scales of bio-inspiration can be formulated in tackling a problem: (i) the organism level, when a specific organism is imitated; (ii) the behavior level, when the behavior of the organism in a larger context is mimicked; (iii) the ecosystem level, if the whole context serves for bio-inspiration [2]. The majority of current research works in the structural engineering realm are focused on form, shape, and material, i.e., the first level. For example, an overview of bio-inspired vibration isolation systems is reported in [3]. Comprehensive reviews devoted to different aspects of bio-inspired forms and materials including dynamic behavior, energy absorption, and advanced material can be found in [4,5,6], whereas some studies, generally not in the structural engineering field, are devoted to bio-inspired algorithms, an exclusive focus on nature as a source of structural design, i.e., design philosophy, is seldom reported.

With the increase in the popularity of bio-inspired solutions, different related but not completely alike terms have emerged: bionics, biomimetics, biomimicry, etc. These concepts are more or less overlapping and the borders are not always very clear. However, they focus on different aspects and levels of bio-inspired solutions with different origins. In this study, such differences are ignored, and the terms bio-inspired science or bio-inspired solutions are used as an umbrella term to cover different aspects and levels.

In recent decades, bio-inspired science has become a well-established research area with many applications in different scientific fields, ranging from engineering to the social sciences. However, as mentioned, most of these studies in the structural engineering realm can be categorized into two main classes. The first one relates to the form, shape, and structural configuration adapted, directly or indirectly, from nature. The second one is related to bio-inspired material. There are inherent similarities between these two classes. Actually, the same bio-inspired concepts are applied at different levels; either on the material or structural levels. Anyway, the bio-inspired solutions that are directly used for design, i.e., design philosophy, are really scarce in the structural engineering discipline.

Progressive collapse and structural robustness are among the relevant topics in structural engineering [7,8,9,10]. Different methods for the design against extreme events to ensure structural robustness are discussed by Starossek [11]. Among them, the alternate load path (ALP) method, which consists of providing alternative solutions for transferring the forces from the elevation to the foundation after an initial failure, is well-accepted and widely used both in research and practice. Whereas the ALP method is the main code-based method to ensure structural robustness, the compartmentalization approach [11] (i.e., creating deliberate discontinuities in the structural scheme to control the propagation of damage), is also adopted in both research and practice, especially for long-span structures, namely bridges. The process of conceiving methods for ensuring enough robustness to a structure and preventing local damage from progressing into the collapse of the whole building followed, so far, heuristic approaches mainly based on the observation of previous accidents. The analogy between current robustness approaches and nature’s solution has not been highlighted, so far.

In addition to the classic approaches, new horizons are also emerging. Among them, the digital twin concept, i.e., a digital representation of a system that updates from real-time data, is striking [12,13]. This growth is largely driven by advances in related concepts and technologies, namely artificial intelligence, machine learning, the Internet of things, cloud computing, big data, multi-physical simulation, robotics, 5G, real-time sensors, and quantum computing. Such advances enable the concept to become reality, i.e., allow the dynamic and live monitoring of structures and facilitate interactive structural response based on the acting threat on the system. Indeed, this philosophy, i.e., justified response based on the acting threat, is nature’s outstanding way of protection, defense, and survival. The new technology advancements provide pristine opportunities for bio-inspiration in practice, which was entirely impossible two decades ago.

However, although no direct explicit bio-inspired approach in the development of the current methods for preventing the collapse is traceable, the analogy is insightful. As previously highlighted, the focus on bio-inspired solutions can help in understanding the currently adopted robustness approaches and promote novel solutions for structural design. The structural design concepts that are discussed in this paper, i.e., complexity and compartmentalization, even without focusing on the bio-inspired aspects, are novel ideas. In other words, for the subject of study, even at a pure structural engineering level, we are still struggling with the ideas and concepts, a well-developed and well-accepted framework is not available, and the methodology is not standardized for the design phase, neither for robustness in general nor for complexity and compartmentalization. However, the suggested solutions can be considered as the basis for future advances to develop practical frameworks for next-generation bio-inspired structural design under extreme loading conditions. This study focuses on the bio-inspired approaches that can be used for the robustness design of engineering structures, especially civil infrastructures. In this regard, robustness approaches, i.e, compartmentalization and complexity, are discussed in depth and several examples are included. The analogy between nature’s solutions and current engineering solutions is highlighted and under the light of this new insight, current approaches are enriched and novel possible solutions are suggested.

## 2. Design for Robustness

The term robustness is encountered in different scientific disciplines, from engineering to biology. In structural engineering, although a unique definition does not exist [14], the term usually refers to the “insensitivity to the local failure” [11]. In the current design philosophy, the robust structure is thought to be self-sufficient enough to correctly behave to damage from the end of its building to the maintenance and, thus, the structural “organism” is fixed. Although issues on the origin of the damage are still open, structural robustness is not to be confused with resilience, which is related to the use of the structure, e.g., the promptness to resume the activities performed in the building after the damage [9]. Nonetheless, robustness is a major component of disaster resilience [15]. In other words, in most cases, a resilient structure is also robust and it is very unlikely to see a resilient but non-robust system, especially in civil structures. In other disciplines, namely biology, robustness is used to refer to similar concepts [16]. However, the distinctions and overlapping aspects of the robustness and related concepts are not always very clear, since, in biology, the concept is related to canalization, redundancy, stability, and adaptability [17,18].

In the current study, which is devoted to bio-inspired solutions being implemented in structural systems, namely civil infrastructures, robustness is defined as the capacity of the system not to be damaged in a way disproportionate to the initial failure. One must bear in mind that for any possible robustness implementation, precise metrics are needed. To this aim, in structural engineering, several, but not unified, solutions at both local and global levels have been proposed [19,20,21,22]. However, there is still room for improvement and much more effort is needed to develop a general framework (that can be used for different structural systems under different initial local failure regimes) for quantification of structural robustness.

Several methods to ensure structural robustness have a twin in biology. Although other solutions involving strengthening of the structure exist, the present paper emphasizes two alternative approaches for ensuring structural robustness: the compartmentalization and the possibility of rerouting the loads across the structure thanks to the network connection between the elements, i.e., complexity. Such approaches, partially implemented in the structural design as detailed in the specific subsections, are extensively adopted in nature to tackle unexpected and extreme events. That is why in this study the emphasis is put on these strategies with the aim of improving their efficacy within the framework of a bio-inspired robustness-oriented design.

### 2.1. Compartmentalization

Compartmentalization is a design philosophy [9] and has been successfully used in real constructions [11]. Basically, it consists of creating artificial discontinuities (by providing physical discontinuity or changes in the structural property like stiffness and energy dissipation capacity, that lead to discontinuity in the structural behavior under extreme loading conditions) in the structural scheme to avoid the propagation of damage and limit its extent after the occurrence of local failure. The concept of structural compartmentalization has been historically well-known to the builders, and also has been suggested for structures under extreme loading conditions [23]. However, the modern use of this idea for the robustness design of civil structures is related to the several works by Starossek that finally were integrated in [11]. In this study, segmentation is considered a special case of compartmentalization in which the system is physically separated. Obviously, compartmentalization is a more general term that can also be applied to functional and active compartmentalized systems. Compartmentalization is widely used in nature to let a species survive. Sacrifice-for-survival mechanisms can be observed in many living organisms, from the organelle to the organ system, and from the organism to the ecosystem. Among them, autotomy in both plants and animals and hypersensitive response (HR) in plants can be highlighted. At the cell level, programmed cell death (PCD) mechanisms, namely apoptosis, are noteworthy.

**Figure 1:**
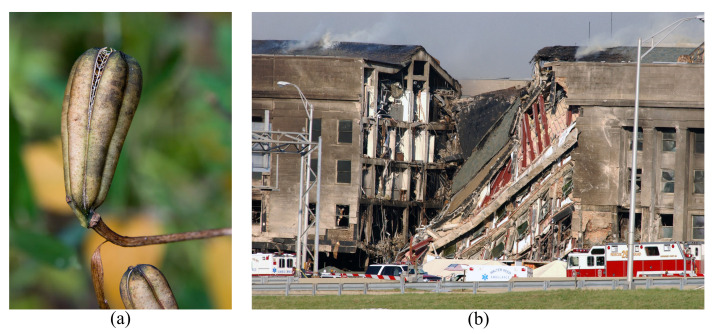
Segmentation concept; (**a**) lilium auratum seed capsule [25], (with no modification under Creative Commons 4.0 license (https://creativecommons.org/licenses/by/4.0/, accessed on 2 February 2023)) and (**b**) the Pentagon building after 9/11 attacks [26].

A well-known example of natural structural compartmentalization can be observed in some plants’ seed pods that are physically segmented (see Figure 1a). A survey with a special focus on biomimetics is reported in [24]. In this case, compartmentalization limits the possible damage to one or a few segments (initial local failure area) and prevents the total destruction of the pod, especially when it is immature. Moreover, compartmentalized seed pods guarantee the uniform distribution of seeds in all directions at release time. This type of physical segmentation can be compared with the construction joints in engineering structures. Such segmentation can save a structure during extreme events such as those observed in the 11 September 2001 attacks on the Pentagon building (see Figure 1b), where expansion joints limited the damage mainly in one segment and prevented the total collapse of the structure [11].

**Figure 2:**
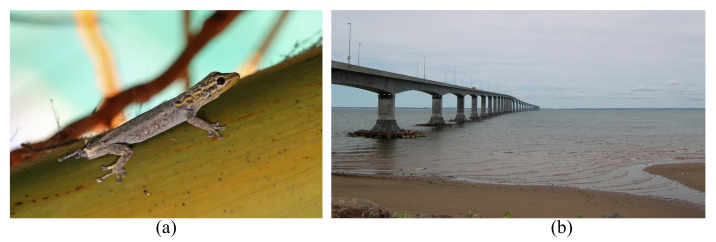
Compartmentalization concept; (**a**) nature: a survived white-headed dwarf gecko with tail lost due to autotomy [33] (taken by Muhammad Mahdi Karim, with no modification under GNU Free Documentation License, Version 1.2) and (**b**) engineering: Confederation Bridge in which a limited collapse is accepted to ensure the structural robustness [34] (with no modification under Creative Commons Attribution 2.0 Generic license (https://creativecommons.org/licenses/by/2.0/, accessed on 2 February 2023)).

Autotomy can be observed in both animals and plants with different levels and mechanisms. With the autonomy, an organism scarifies a body part as a self-defense mechanism to avoid an external threat and subsequently possible death (total failure of the system). One of the best known examples of autotomy is that of the gecko’s tail (see Figure 2a). In this case, the animal employs autotomy to distract predators, but herein, the underlying concept of scarifying the member/part to save the system is noteworthy. However, this mechanism is also observed in other animals, e.g., legs in spiders [27], tails in reptiles [28], arms in brittlestars [29] and even in mammals, as skin in mice [30]. An interesting case from a structural point of view is related to African wood sorrel, in which, when the leaves and flowers of this plant are pulled (i.e., tensile stress) they break easily at their base, leaving the rest of the plant intact [31]. That is in contrast with so-called “strength strategy” adopted, e.g., by woody plants in which the failure occurs in soil-root system [32].

There are two different autotomy mechanisms: first, i.e., true autotomy, in which the animal throws off a part of the body when sufficiently stressed by a threat, but not necessarily with the involvement of mechanical forces. The purpose of such behavior can be either to distract the predators/threat or to release the stress/pain. For example, lizards can contract a muscle to fracture a vertebra [35] under a specific condition (biomimicking interfacial fracture behavior of lizard tail autotomy is discussed in [36]). When spiders are injected in the leg with bee or wasp venom, they can shed this appendage [37] based on the pain level. In the second type, i.e., false autotomy, observed in both animals and plants, the autotomy occurs under direct mechanical stress in a predefined zone as discussed for African wood sorrel [31].

Compartmentalization techniques in the current modern engineering structures are similar to the latter in which controlled failures occur in predefined positions in the structural scheme, namely construction joints, deliberately weak zones, specially designed reinforcement bar configurations and fuse-type elements (see Figure 2b and [10,11]). Although not reported in the literature, theoretically, it is possible to use the “true autotomy” concept in future smart structures. Actually, recent advances in structural engineering, namely digital twin [12,13], can facilitate the application of this concept. Adjustable structural response (based on the acting load/threat) is seldom reported in structural engineering. For example, magnetorheological dampers to mitigate train-induced [38] and rain-/wind-induced [39] vibrations in bridges are noteworthy. The aforesaid example of the spider injected with wasp venom [37] can be revisited here. The structural changes in these methods (e.g., adjusting the stiffness in specific points and directions that can modify the dynamic property of the system) are far less than what is actually needed for active compartmentalization, in which almost complete segmentation is required. However, tracing recent advances in both monitoring science and construction techniques guarantees that the “true autotomy” concept can be used in future modern structures. Nature also provides more interesting ideas, for example, a delayed response in *Verbascum sinuatum* (wavyleaf mullein) is also inspiring [31]. The idea is useful, for example, for allowing evacuation before the controlled partial collapse in the compartmentalization strategy.

**Figure 3:**
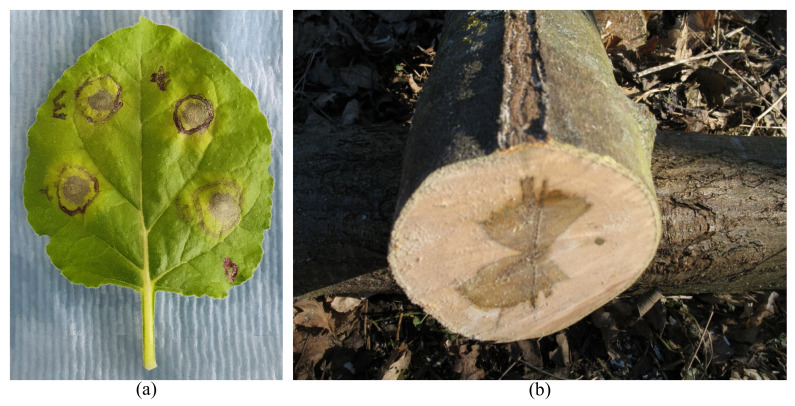
Compartmentalization by isolation in nature; (**a**) lesions caused by the plant hypersensitive response [46] (with no modification under the Creative Commons Attribution-Share Alike 4.0 International license (https://creativecommons.org/licenses/by-sa/4.0/, accessed on 2 February 2023)) and (**b**) compartmentalization of decay in Norway maple tree [47] (with no modification under the Creative Commons Attribution-Share Alike 3.0 Unported license (https://creativecommons.org/licenses/by-sa/3.0/, accessed on 2 February 2023)).

Hypersensitive response (HR) in plants is another situation in which the compartmentalization concept is used in a living organism. HR is characterized by the rapid death of cells in the local region surrounding a threat (usually pathogens) to prevent the spread of the problem to other intact parts of the plant (see Figure 3a). Compartmentalization of decay (damage) in trees (CODIT) is also noteworthy here [40,41]. When a tree is wounded under a specific threat, the damaged region does not usually heal or replace, in contrast to what usually occurs in animals. Alternatively, trees isolate the damaged parts by producing new tissue around the damaged region, creating a protective boundary and isolating the damaged tissue due to decay or infection (see Figure 3b). The concept can be adopted for the compartmentalization of affected areas in corrosion, aging, and chemical attack in concrete and steel. Heretofore, the studies are usually limited to non-structural levels.

There are several other situations where sacrifice-for-survival mechanisms act to save the organisms. An example of such a mechanism is reported in a root stem cell niche subjected to chilling stress [42]. Programmed cell death [43], namely apoptosis, shows interesting and useful characteristics. In vertebrates, necroptosis [44] as a PCD mechanism can also be considered, where cell suicide in a programmed fashion aids in defense against pathogens. Two mechanisms can be observed in apoptosis: the “intrinsic pathway”, in which the cell kills itself because it senses stress, and the “extrinsic pathway”, in which the cell kills itself because of *signals* from other cells [45]. In currently engineered compartmentalization, the compartmentalized region is usually within a damaged area or in its vicinity. On the other hand, inspired by the PCD, cases can be defined where compartmentalized regions are activated based on the damage progress and threat situation. With ongoing advances in digital twin and related concepts, there are reasons to be optimistic that a real-time digital replica of the system will soon be possible (actually such techniques with some limitations are already used in special structures [12,13]). Such a revolutionary concept, plus the burgeoning applications of artificial intelligence and machine learning, allows the prediction of structural response and determines critical scenarios faster than acting threats. Thenceforward, the most suitable region (from global structural integrity, economical loss, or a life-saving point of view) can be compartmentalized. Future smart structures, hypothetically, can monitor the threat progress (say for example fire) and determine the damage level (which members and to what extend *are affected* and *will be affected*) to *predict* and *decide* about compartmentalization schemes, that can be far from the direct damage region and even in the intact parts of the system to increase efficiency and decrease overall loss.

Despite compartmentalization effectiveness, it is very costly and it can be considered only after other defense measures have failed. In nature, compartmentalization mechanisms are usually the last line of defense. A similar concept is already used in structural engineering to avoid progressive collapse, in which compartmentalization is only considered for very large initial failure or when the existence of ALPs cannot be guaranteed [10].

**Figure 4:**
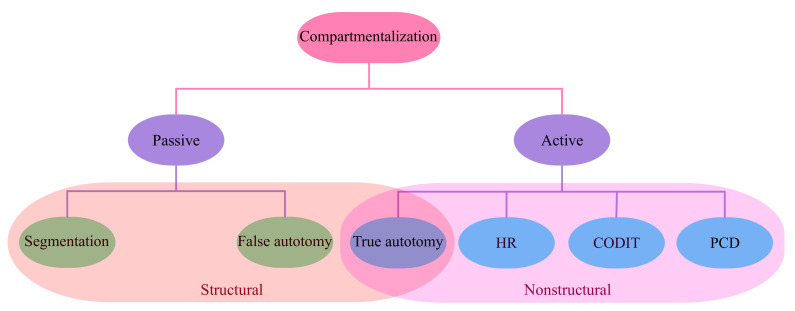
Classification of different natural compartmentalization phenomena. HR, CODIT, and PCD stand for *hypersensitive response*, *Compartmentalization of decay (damage) in trees*, and *programmed cell death*, respectively.

In general, natural compartmentalization phenomena are either active or passive. In the active form, namely true autotomy in the spider leg, the system decides about the segmentation necessity and appropriate time based on the threat level, therefore, compartmentalization can happen even before any physical damage. On the other hand, in false autotomy, for example for African wood sorrel, compartmentalization is achieved in a predefined weak region and then activated under specific mechanical stresses. Alternatively, compartmentalization can be categorized either as structural or non-structural, whereas in the former, some mechanical properties (special configuration in geometry and/or material) allow the compartmentalization at extreme conditions; in the latter, namely HR and CODIT, this is achieved using the biochemical changes. Figure 4 shows this classification.

### 2.2. Complexity

Complexity is the characteristic of systems in which specific properties are the result of the mutual participation of the elements of the system and, thus, the whole is not the mere sum of its components. There is no unique and well-accepted definition of complexity and the published definitions usually depend on the topic and type of the system in which the term is applied. Complexity characterizes the behavior of a system whose components interact in multiple ways and follow local rules, meaning there is no reasonable higher instruction to define the various possible interactions [48]. Similarly, it can be stated that a system’s behavior is not the simple sum of the behavior of its components [49]. Weaver drew a distinction between “disorganized complexity” and “organized complexity” based on the number of the parts and interactions between them [50].

The emergence of behaviors from the arrangement of elements, each of which acts in a separate way, is typical of connected systems [51]. In the civil engineering realm, a complex structure is one that cannot be reduced to a simple scheme without losing important aspects of the structural behavior [49]. Complexity is not a well-documented approach for increasing structural robustness and the research works on this topic are mainly limited to the handful of papers [49,52,53], whereas a limited number of studies have focused on the quantification of the structural complexity [49,52,53]. To date, no uniform framework for distinguishing between complex and non-complex structure exists.

**Figure 5:**
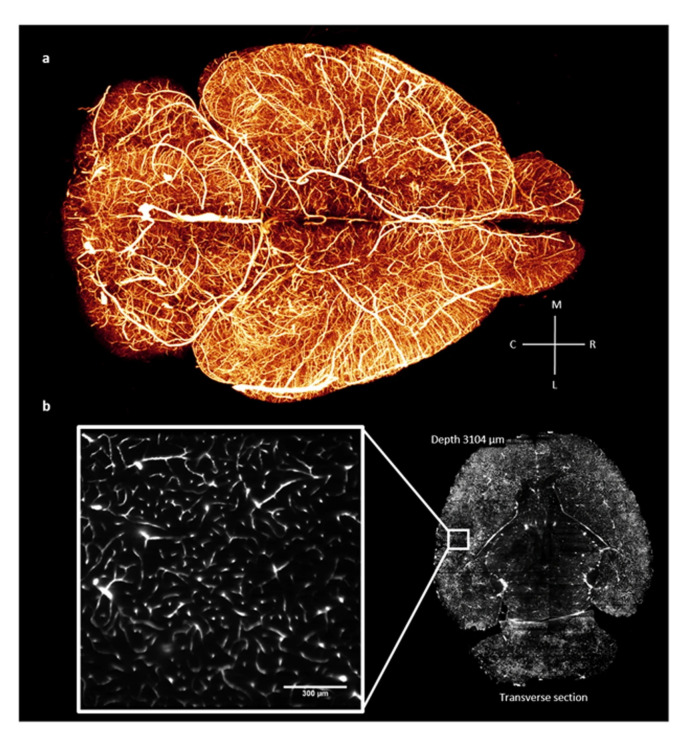
Whole mouse brain vasculature tomography; (**a**) 3D rendering and (**b**) single frame from a stack at original resolution, as reported in [57], (with no modification under Creative Commons 4.0 license (https://creativecommons.org/licenses/by/4.0/, accessed on 2 February 2023)).

Complexity is among the main approaches that can be found in live organisms to ensure system robustness. The concept of complexity is similar, but not equal, to redundancy and ALP. Complexity is more a matter of interaction between different systems and sub-systems, which mutually influence each other when responding to an input. However, in certain structural systems, say precast reinforced concrete structures, redundancy can be considered among the few possible strategies for providing robustness to the system [54]. In the concept of ALP, the system shows different responses to different initial failure scenarios, but, a complex system is *insensitive* to the initial failure (regardless of the size and location of the initial failure), and therefore, is a robust system (an example of a natural complex system, i.e., mouse brain’s vascular network, is shown in Figure 5). In another word, as argued by Kitano [55], robustness is a fundamental feature of evolvable complex systems. As another example, simple bacteria with several hundred genes require carefully controlled environments, whereas others, with ten times the number of genes, can survive when subjected to extreme conditions [56].

Complex systems are usually redundant. Redundancy is a universal property of the nervous systems from lobster stomatogastric ganglion [58] to the human brain [59]. Three sub-concepts of redundancy in the nervous system including sloppiness, compensation, and multiple solutions are suggested and discussed in [60]. Considering that no effective classification of structural complexity is suggested so far, such a functional categorizing is inspiring. At the ecological level, functional equivalence can be considered in which multiple species can share similar, or even identical, roles in an ecosystem [61]. Several examples of this type of redundancy in the complex systems, from plant–pollinator relationships [62] to plant–animal seed dispersal mechanisms [63] can be mentioned. In structural engineering, heretofore, no classification referring to the involved mechanisms is suggested. However, inspired by nature, namely from functional equivalence and biodiversity, the involving mechanisms in a complex system can be classified based on form and function.

Analyzing biology and complexity, Carlson and Doyle [56] highlighted that the highly optimized tolerance (HOT) conceptual framework well describes the ability of microorganisms to be extremely robust. This ability is the result of millions of years of evolution that created a biological system that is well-structured, heterogeneous, and self-dissimilar (i.e., different patterns are observed at different scales). This allows the system to adapt to large events, with an intrinsic robustness and the ability to respond differently, but with an inherent fragility to local failures, the so-called “robust, yet fragile” consideration. The HOT framework contrasts with the self-organized criticality (SOC) model which argues that living organisms show a robust behavior by changing from one steady state to the other, and not by maintaining a given state [16]. To provide such a property, the internal configuration of the system should be generic and self-similar. Although the two models try to describe biological complexity and robustness, suggestions for engineered systems can be drawn. One of the key points that differentiate between living and engineered entities is the possibility for the former to evolve over time. Usually, artificial systems, say civil structures, are designed and built not to change. The possibility to adapt, as inspired by nature, is a key point in the design of bio-inspired robust structures.

**Figure 6:**
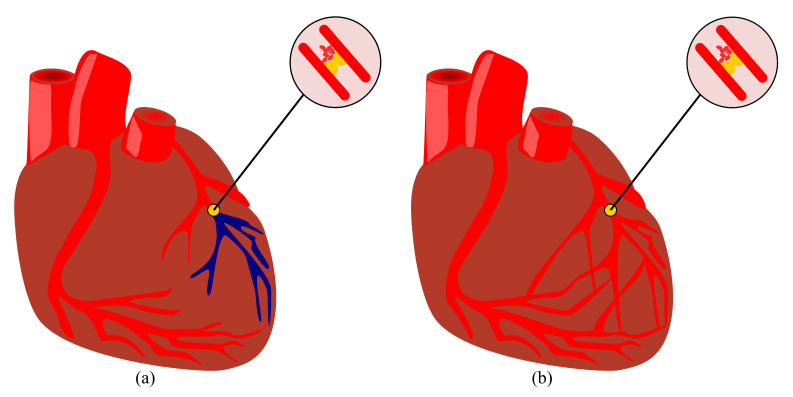
Schematic drawing of the coronary artery circulation (**a**) without and (**b**) with collateral circulation, based on the concept reported in [65].

As mentioned, the ALP method is the main design approach in current research and practice in structural engineering. However, in the current study, ALP is discussed as a subset of complexity. This reflects the fact that complexity approaches can be found in living organisms that ensure system robustness. ALP can be considered as an *engineered equivalent* of complexity in human-made systems. In the ALP approach, the ability of the structural system after an initial local failure, namely a member loss, is examined, whereas there is no evidence that this approach was developed by bio-inspiration, a similar concept adopted in natural systems for millions of years. A clear example of the ALP concept can be observed in collateral circulation (see Figure 6). Collateral circulation is the alternate circulation around a blocked artery or vein via alternative paths. These alternate paths can be the existing vessels or newly developed ones. Several examples of both situations can be observed in different parts of the human body, namely the brain, heart, and kidneys [64]. A complete analogy can be observed in engineering structures after initial failure in which alternate load paths activate to prevent the total collapse of the structural system.

**Figure 7:**
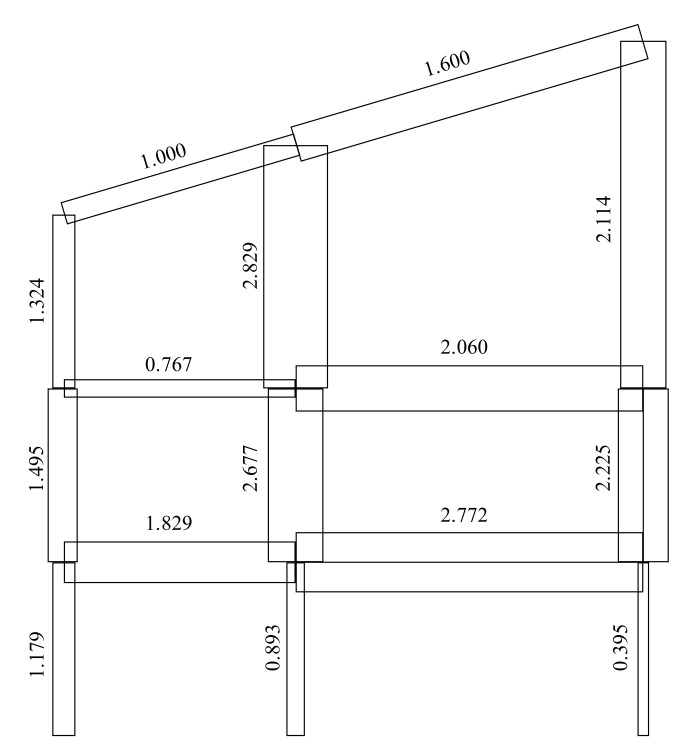
Sketch of a structure subjected to vertical and lateral loads on nodes. The size of the elements results from the maximization of the normalized structural complexity index [67].

Following biological insights, two possible trends emerge. On one side, there are structural designs that foster some specific types of damage tolerance and try to uniform the response of the system to the threat: this is the case, for example, of those schemes in which the structural complexity with a defined loading scheme is maximized [66]. As an example, Figure 7 illustrates a frame structure subjected to vertical and lateral loads (equal magnitude) on nodes. The size of the elements ensures maximization of the normalized structural complexity index (NSCI) of the system; hence, the effects of a local element removal are similar wherever the location of the damage [67]. Nevertheless, it is theoretically possible to fully implement the natural strategies for ALP on future smart structures, i.e., structures that are able to modify their stiffness, connections, and constraints depending on the load types and intensity acting on them.

Besides, such schemes are prone to failure if the loading changes. On the other side, there are structures that are designed to resist specific threats, only, e.g., column removal at the bottom level, for all the possible combinations of live and dead loads. As highlighted in biological robustness studies, there is a balance between robustness, fragility, performance, and resource demand that rules the shape of the systems [55].

ALP strategy can also be implemented by adopting ultra-specialized materials. A clear example from nature comes from the analysis of the local failure of spider webs. Spider webs are masterpieces of natural structural engineering [68]; millions of years of evolution shaped them in order to achieve a desired optimized functionality, i.e., the capture of prey using a minimum amount of silk [69]. Deeply analyzing a spider web, its structural integrity is guaranteed by the stiff behavior of silk under small deformation before the yield point. As proven, the web structural performance is dominated by the properties of the stiffer and stronger radial dragline silk, suggesting that the spiral threads play non-structural roles, i.e., capturing prey [70].

**Figure 8:**
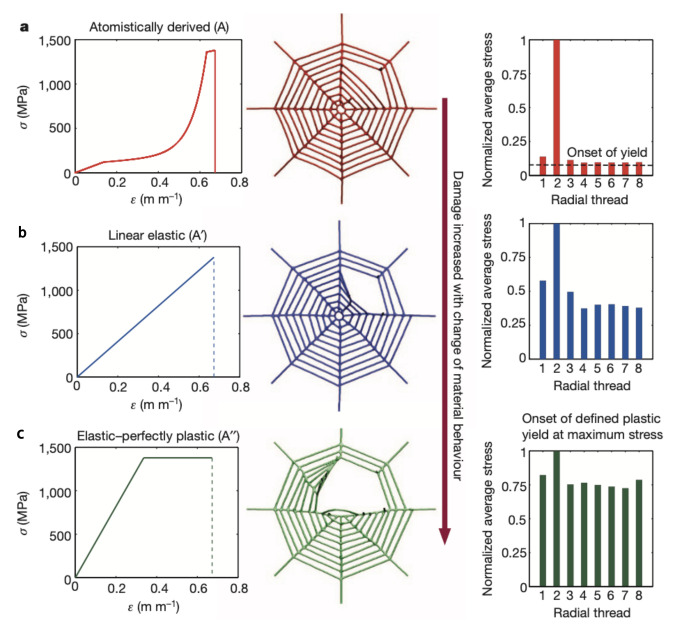
Different behaviors depending on material properties of spider web from top to bottom; (**a**) the real material properties, (**b**) linear elastic behavior, and (**c**) elastic–perfectly plastic behavior. The damages after similar removal are plotted, as reported in [71], reprinted with permission.

Cranford et al. [71] simulated the response of spider webs made of different types of fibers with completely different mechanical behaviors (Figure 8). Model (a) refers to the stress–strain behavior of the dragline silk from the species *Nephila clavipes*. Four distinct regimes characterize the behavior: an initial linear part governed by stretching, an unfolding of the protein domain resulting in a softening that continues with a stiffening regime, and, finally, a stick-slip deformation. Models (b) and (c) refer to idealized engineered materials with linear elastic and elastic–plastic behavior, respectively.

The initial damage is represented by the cut of a radial element. It results that any change in deformation behavior and web damage would be a direct result of differences in the stress–strain behavior of the fibers. In the case of a web composed of natural dragline silk, all radial threads partially contribute to loading resistance. The fact that the material suddenly softens at the yield point ensures that the transfer is limited to the loaded radial thread, which begins to stiffen. In the linear elastic model, the loaded radial threads are subjected to the majority of the load. In this way, the adjacent radial threads bear a higher fraction of the ultimate load, which results in a greater delocalization of damage after the failure. With the elastic–plastic material, the perfectly-plastic behavior of the radial element enhances load distribution throughout the structure and it greatly increases the damage zone.

## 3. Discussion and Conclusions

There is an increase in bio-inspired research and its application in civil and structural engineering. However, as reviewed, the available literature is mainly devoted to bio-inspired form, shape, and material, not to structural design philosophy. This paper tries to put forth some novel bio-inspired design strategies to ensure the robustness of civil structures and infrastructures. In this regard, nature’s solutions are comprehensively reviewed and various examples are provided. Table 1 summarizes nature’s solutions and their possible engineering equivalents for robustness design. Among the proposed solutions, the emphasis is put on two alternative but complementary solutions, i.e., complexity and compartmentalizing. Different aspects of these novel concepts are scrutinized and several suggestions are proposed for future smart structures.

It should be noted that the application of the suggested new concepts will be facilitated with more advances in other fields, namely, construction science, robotics, real-time sensor network, and digital twin. In the both mentioned classes of structural robustness strategies, i.e., complexity and compartmentalization, modifications of the stiffness and connectivity between members/parts is required. In the former, the stiffness of the member at the local level and the connections between the members at a global level (that indicate the global stiffness and strength of the system) can be manipulated to maximize the complexity of the system at different loading regimes (or acting threat, i.e., various initial local failures). For the latter (compartmentalization), the possibility of active discontinuity at different levels is favorable.

For the materialization of such adjustability and adaptability, two important steps still need to be taken. (i) The live monitoring and analysis of the structure, which allow the live assessment of structural response, and determine the best possible “changing scenario”, in terms of removal, discontinuity, and/or adjustment of the stiffness and energy dissipation capacity, are necessary. To this aim, some fundamental progress, namely digital twin, is already achieved. However, more advances are still required. In addition, (ii) the next-generation smart structures should be able to change their stiffness and connectivity. This ability is very limited in the existing modern structures, but is absolutely necessary to realize the concepts suggested in the paper. Recent advances in construction science and robotics serve this aim. It is anticipated that such a technique will first appear in space and military applications and then will spread to critical infrastructures. Thitherto, we need to develop ideas and test the concepts, and this study is dedicated to that purpose.

Two main classes for bio-inspired robustness design of civil structures and infrastructures, namely compartmentalization and complexity, are observed and discussed. Analogy with the current design approaches is demonstrated and possible improvements are highlighted. For compartmentalization, new bio-inspired concepts, namely (i) delayed compartmentalization, (ii) active compartmentalization, and (iii) compartmentalization in intact part are suggested. Structural complexity, as a bio-inspired robustness technique, is suggested and discussed. Recent progress in monitoring techniques and burgeoning construction advances will enable structural scientists to mimic nature more closely, and the future modern and smart structures can be “live” from material level to global system level.

## Figures and Tables

**Table 1:** Natural solutions and their analogy in structural engineering for robustness.

Nature Solution	Structural Engineering Solution
Segmentation	Construction joints
False autotomy	Structural segmentation/compartmentalization
True autotomy/HR/ PCD	Possible use in next generation active robustness techniques
CODIT	Possible use in next generation self-compartmentalizing concrete-corrosion in steel
Collateral circulation	ALP method
HOT	Structural complexity

## Data Availability

Not applicable.
